# Clinical implications of a history of stealing on psychiatric disorders in children and adolescents

**DOI:** 10.1371/journal.pone.0237906

**Published:** 2020-08-27

**Authors:** Yoshinori Sasaki, Tatsuhiko Yagihashi, Mari Kasahara, Masahide Usami, Toshiaki Kono, Takayuki Okada

**Affiliations:** 1 Department of Psychiatry and Behavioral Science, Tokyo Medical and Dental University Graduate School, Tokyo, Japan; 2 Department of Child and Adolescent Psychiatry, Komagino Hospital, Hachioji, Tokyo, Japan; 3 Department of Child and Adolescent Psychiatry, Kohnodai Hospital, National Center for Global Health and Medicine, Ichikawa, Chiba, Japan; 4 Department of Community Mental Health and Law, National Institute of Mental Health, National Center of Neurology and Psychiatry, Kodaira, Tokyo, Japan; University of the West Indies at Saint Augustine, TRINIDAD AND TOBAGO

## Abstract

**Background/Aim:**

To our knowledge, no case–control study has investigated the relationships between stealing, clinical implications, and psychiatric diagnosis among child and adolescent psychiatric patients with or without a history of stealing. Thus, the associations between child and adolescent psychiatric disorders and a history of stealing remain unclear. Therefore, the aim of the present study was to evaluate the relationships between stealing, clinical implications, and psychiatric diagnosis among child and adolescent psychiatric patients with or without a history of stealing.

**Methods:**

In this retrospective case–control study, the proportions of clinical implications among child and adolescent psychiatric patients with and without a history of stealing were compared. Data regarding age, sex, primary diagnosis, junior high school student or not, both father and mother are the caregivers or not, family history, abuse history, school refusal, depressive state, and obsessive–compulsive symptoms were retrieved from medical records. Participants consisted of Japanese junior high school students and younger patients (maximum age, 15 years) at the first consultation. All patients were examined and diagnosed by psychiatrists according to the Diagnostic and Statistical Manual of Mental Disorders, Fourth Edition, Text Revision, or the Fifth Edition. Stealing was reported by the patients or caregivers to the psychiatrist, or the psychiatrist had inquired about a history of stealing at the first consultation.

**Results:**

Among 1972 patients who consulted the clinic, at the first consultation, 56 (2.84%) had a history of stealing (cases), and 1916 (97.16%) did not (controls). Multivariate logistic regression analyses revealed that the proportions of males, junior high school students, abuse history, autism spectrum disorder (ASD), and conduct disorder were significantly higher, and the proportions of adjustment disorders and school refusal were significantly lower in cases than in controls. The multivariate adjusted odds ratio increased further when the two factors were considered together, such as ASD with abuse history and attention deficit–hyperactivity disorder (ADHD) with abuse history.

**Conclusions:**

Children with a history of stealing were more likely to be diagnosed with ASD or ADHD with abuse history. Child and adolescent psychiatric outpatients with a history of stealing were more likely to be older and male. Our study should be understood without prejudice because this study is reporting associations, not causality. Therefore, a prospective study to investigate causality among ADHD, ASD, abuse history, and stealing is needed. If ADHD and ASD with abuse history can be correlated to a history of stealing, interventions can be more effective by understanding the mechanisms underlying these connections.

## Introduction

Juvenile delinquency is an important public health concern that jeopardizes not only the safety of the juvenile but also the well-being of society as a whole [1]. Adolescent antisocial behavior widely affects society in a penetrating manner, which involves victimization and distress to individuals, impairment of life opportunities, and staggering costs to society [2, 3]. In Japan, theft accounts for approximately 60% of arrests and a similarly high proportion of juvenile delinquencies due to penal code offenses in 2016. Of those arrested, shoplifting accounts for 36.8% of thefts [4]. According to the Diagnostic and Statistical Manual of Mental Disorders, Fifth Edition (DSM-5), kleptomania is defined as a pathologic stealing disease. However, the prevalence of kleptomania in the general population is rare, at approximately 0.3% to 0.6% [5] and the average age of onset for kleptomania has been reported to be 18.75 years [6]. Stealing in children and adolescents is not attributed to kleptomania.

The frequency of stealing increased from 12 to 18 years old [7] and adult shoplifters were classified into several types according to the reason and purpose for stealing [8]. Associations between stealing and psychiatric disorders was reported in adults. Stealing was strongly associated with impulse-control, substance-related and addictive disorders [9, 10]. With regard to sex differences, women were likely to be diagnosed with mood disorders and antisocial personality disorders, whereas men were likely to be diagnosed with generalized anxiety disorder [11].

Some research has focused on the relationship between stealing and psychiatric disorders in young people. Urges to steal in university students was associated with bipolar disorder and multiple impulse control disorders [12]. Among high school students, 15.2% reported a history of stealing. Poor grades, alcohol and drug use, regular smoking, sadness, hopelessness, and other antisocial behaviors was associated with stealing [13].

In a case–control study of child and adolescent psychiatry outpatients, Heath and Kosky reported that patients with a history of stealing exhibited symptoms of depression or anxiety [14]. However, this study compared those with a history of stealing and aggressive behaviors, but not the patients without a history of stealing. In a cohort study of Finnish males with a history of stealing until 25 years of age, psychiatric diagnoses were investigated. Stealing was associated with completed suicide or severe suicide attempts requiring hospitalization [15]. However, this study considered psychiatric diagnoses alone after 18 years of age. In a prospective cohort study to assess neurocognitive functions of males with a history of stealing, Barker found that verbal ability was positively associated with an increased incidence of stealing throughout adolescence [16]. This study did not evaluate clinical implications.

To our knowledge, no case–control study has investigated the relationships between stealing, clinical implications, and psychiatric diagnosis among child and adolescent psychiatry outpatients with and without a history of stealing. Thus, the association between psychiatric disorders in child and adolescent patients with a history of stealing remains unclear. Therefore, the primary aim of the present study was to evaluate the relationships between stealing and clinical implications among child and adolescent psychiatric patients with and without a history of stealing.

The main hypothesis of this study was that stealing is not associated with a single cause but rather with various biological factors, including developmental and psychological disorders, as well as the nurturing environment, which may lead to stealing. As a secondary hypothesis, since people who steal were reportedly more likely to engage in stealing as they get older, we hypothesized that patients with a history of stealing would tend to be older than those without.

These hypotheses indicate that clinical examinations of patients with a history of stealing should evaluate not only the underlying causes of stealing but also the background data. If these hypotheses are linked to detailed clinical implications, more specific preventive and treatment programs can be implemented.

## Methods

### Study design and setting

A retrospective case–control design was used to evaluate the relationships between stealing, clinical implications, and psychiatric diagnosis among child and adolescent psychiatric outpatients with a history of stealing as compared with those without.

In line with the case–control design, the study participants were allocated to one of two groups: those with a history of stealing (cases) and those without (controls). The analyzed data included age, sex, primary diagnosis, junior high school status, both the father and mother are the caregivers or not, family history, abuse history, school refusal, depressive state, and obsessive–compulsive symptoms.

The study protocol was approved by the Medical Research Ethics Committee of Tokyo Medical and Dental University (Tokyo, Japan) and the local Ethics Committee of Komagino Hospital (Tokyo, Japan) and conducted in accordance with the tenets of the Declaration of Helsinki. Informed consent was obtained from all subjects in accordance with Ethical Guidelines for Medical and Health Research Involving Human Subjects of Japan. The guidelines state that “It is not always necessary to obtain informed consent from study participants. However, researchers must publish information on the implementation of the study, including the purpose of the study” for observational studies only using past clinical records and not human tissue samples. The purpose, methods, inquiry of the study, and how to refuse participation were posted in the hospital’s outpatient clinic. In addition, the research data were anonymized because patient correspondence was unnecessary throughout the study period. The data from this study may contain potentially identifiable patient information, and sharing the data is restricted by the Medical Research Ethics Committee of Tokyo Medical and Dental University and the local Ethics Committee of Komagino Hospital based on Ethical Guidelines for Medical and Health Research Involving Human Subjects of Japan. To access the data, please contact Medical Research Ethics Committee of Tokyo Medical and Dental University at syomu1.adm@tmd.ac.jp.

### Recruitment and participants

The study participants consisted of Japanese junior high school students and younger children who visited the Department of Child and Adolescent Psychiatry of Komagino Hospital for the first time between August 2010 and March 2017. The junior high school students were aged 12 to a maximum of 15 years. As the beginning of puberty, we accounted for whether the participants were junior high school students or not. The study participants resided in Hachioji City and the surrounding areas. Hachioji is a city with a fully developed residential area and a center of education that is situated in the western part of Tokyo, approximately 40 km from the metropolitan area. In March 2019, the population of Hachioji was estimated to be 561,407. Psychiatrists specializing in child and adolescent psychiatry completed the intake interview forms for all patients, which included the demographic characteristics of caregivers, clinical features, and diagnoses based on psychiatric interviews conducted during the first consultation. These interviews were conducted using unstandardized forms addressing 32 past or present clinical symptoms (hallucination, depression, etc.) and psychosocial states (caregivers, abuse history, etc.) which were based on DSM-5 classification “other conditions that may be a focus of clinical attention” [5]. Because Komagino Hospital worked with the local child protection service, information pertaining to abuse history was often available prior to the first consultation. Moreover, psychiatrists in charge interviewed parents and patients separately to detect abuse, depending on the situation. With these efforts, we detected abuse as early as possible and prevented missed abuse. Patients with moderate to severe intellectual disability, organic brain disease, drug-induced psychiatric disease, traumatic brain injury, and genetic syndromes were referred to other medical institutions and excluded from this study. All patients were examined and diagnosed according to the Diagnostic and Statistical Manual of Mental Disorders, Fourth Edition, Text Revision (DSM-IV-TR) or DSM-5 [5, 17]. All diagnoses were made by psychiatrists. Meetings to discuss the accuracy of the diagnosis and the possibility of abuse history were conducted as needed. For patients with comorbidities, the diagnosis that could best explain the condition was used as the primary diagnosis. The definition of stealing was based on reports of a history of stealing from patients and caregivers to the psychiatrist at the first consultation. The caregiver's report, not the patient's report was likely to be the definitive information about stealing. After listening to the narratives of them, the psychiatrists decided whether or not their behavior was considered as stealing. In several cases, stealing inside the house meant spending money without the caregiver’s permission, and stealing outside the house meant shoplifting and pickpocketing. As an example, we did not judge their behavior as stealing solely because they borrowed clothes and a bicycle, ate something secretly when they were hungry without the caregiver’s permission. School refusal is when a student has been absent from school for 30 days or more during the school year because of refusal to attend classes due to any psychological, emotional, physical, or social factor or background [18]. The involvement of the mother and father in caregiving was investigated to evaluate the nurturing environment. Family history included whether anyone in the biological family consulted a psychiatrist, was hospitalized, or committed suicide due to a psychiatric disease. Child and adolescent psychiatrists evaluated the status of depressive and obsessive–compulsive symptoms. Clinical data without personal information were used for analysis.

### Case–control design

Cases (stealing group) included patients with a history of stealing who were interviewed by a psychiatrist at the first consultation. Controls (non-stealing group) included patients without a history of stealing at the first consultation.

### Statistical analysis

Fisher’s exact test was used to compare proportions of binary variables between the two groups (Table 1), and the Mann–Whitney U test was used to compare continuous variables between the two groups. Multivariate logistic regression analysis was performed to calculate the odds ratio (OR) and 95% confidence interval (CI) (Tables 2–4). Possible confounding factors selected from previous studies and medical viewpoints, as described in the introduction, were included for multivariate logistic analysis. Following consultation with a statistician, five variables were assessed for the total number of cases. All statistical tests were two-tailed, and a probability (*p*) value of <0.05 was considered statistically significant. Analyses were performed using the Easy R Package version 1.40 [19].

**Table 1:** Clinical backgrounds of the study participants.

Parameter	Participants with a history of stealing (cases) % (n = 56)	Participants without a history of stealing (controls) % (n = 1916)	OR	95% CI	*p*
Age (mean ± standard deviation)	12.39 ± 1.93	11.03 ± 2.92	-	-	<0.01
Male (%)	73.2 (n = 41)	55.9 (n = 1071)	2.16	1.19 to 3.92	<0.05
Junior high school student (%)	66.1 (n = 37)	46.1 (n = 884)	2.27	1.30 to 3.98	<0.01
Fathers and mothers as caregivers (%)	53.6 (n = 30)	68.2 (n = 1306)	0.54	0.32 to 0.92	<0.05
Family history (%)	50.0 (n = 28)	52.1 (n = 998)	0.92	0.54 to 1.57	NS
abuse history (%)	35.7 (n = 20)	13.2 (n = 253)	3.65	2.08 to 6.41	<0.01
School refusal (%)	25.0 (n = 14)	40.7 (n = 779)	0.49	0.26 to 0.90	<0.05
Depressive state (%)	21.4 (n = 12)	22.1 (n = 424)	0.96	0.50 to 1.83	NS
Obsessive–compulsive symptoms (%)	1.8 (n = 1)	6.5 (n = 125)	0.26	0.04 to 1.90	NS
**Primary diagnosis**					
ADHD	32.1 (n = 18)	18.2 (n = 349)	2.13	1.20 to 3.77	<0.05
ASD	28.6 (n = 16)	16.2 (n = 310)	2.07	1.15 to 3.75	<0.05
Conduct disorder	12.5 (n = 7)	0.3 (n = 6)	45.5	14.7 to 140.0	<0.01
Intellectual disability (mild)	7.1 (n = 4)	4.8 (n = 92)	1.53	0.54 to 4.31	NS
Adjustment disorder	5.4 (n = 3)	22.4 (n = 429)	0.20	0.06 to 0.63	<0.01
Reactive attachment disorder	5.4 (n = 3)	1.3 (n = 25)	4.28	1.25 to 14.6	<0.05
Paraphilic disorders	1.8 (n = 1)	0 (n = 0)	-	-	<0.05
Posttraumatic stress disorder	1.8 (n = 1)	1.4 (n = 27)	1.27	0.17 to 9.53	NS
Anorexia nervosa	1.8 (n = 1)	0.6 (n = 12)	2.88	0.37 to 22.6	NS
Intellectual disability (borderline)	1.8 (n = 1)	0.4 (n = 8)	4.34	0.53 to 35.3	NS
Kleptomania	1.8 (n = 1)	0 (n = 0)	-	-	<0.05
	Total N = 56				

“-” indicates that the OR and 95% CI could not be calculated for n = 0.

The “primary” diagnosis was the diagnosis that could best explain the condition.

**Table 2:** Multivariate logistic regression analyses of demographic and clinical characteristics.

Parameter	Multivariate adjusted OR	95% CI	*p*
Male (%)	2.38	1.29 to 4.39	<0.01
Junior high school student (%)	3.40	1.89 to 6.14	<0.01
Fathers and mothers as caregivers (%)	0.87	0.47 to 1.60	NS
abuse history (%)	3.73	1.95 to 7.11	<0.01
School refusal (%)	0.35	0.18 to 0.66	<0.01

Each parameter was the result of the adjustment for the other four parameters.

**Table 3:** Multivariate logistic regression analyses for diagnosis.

Parameter	Multivariate adjusted OR	95% CI	*p*
ADHD	1.87	1.00* to 3.49	NS
ASD	2.02	1.09 to 3.74	<0.05
Conduct disorder	49.5	14.9 to 164	<0.01
Intellectual disability (mild)	1.46	0.50 to 4.25	NS
Adjustment disorder	0.20	0.06 to 0.66	<0.01
Reactive attachment disorder	2.14	0.56 to 8.20	NS

*The value was increased to 1.00 after rounding up of the third decimal place.

After adjusting for four parameters (sex, proportion of junior high school students, abuse history, and school refusal).

**Table 4:** Multivariate logistic regression analyses of ADHD and ASD with abuse history.

Parameter	Multivariate adjusted OR	95% CI	*p*
ADHD with abuse history	7.81	3.21 to 19.0	<0.01
ASD with abuse history	4.46	1.48 to 13.4	<0.01

After adjusting for two parameters (sex and proportion of junior high school students).

## Results

### Participants and descriptive data

Of the 1972 patients who consulted the Department of Child and Adolescent Psychiatry, Komagino Hospital, from August 2010 to March 2017, 56 (2.84%) had a history of stealing, and 1916 (97.16%) did not. Patient demographics are described in Table 1.

Table 2 presents the results of multivariate logistic regression analyses between cases and controls adjusted for sex, junior high school student status, proportion of patients with fathers and mothers as caregivers, abuse history, and school refusal. Each parameter was the result of the adjustment for the other four parameters.

Table 3 presents the results of multivariate logistic regression analyses between cases and controls for a diagnosis of ADHD, autism spectrum disorder (ASD), conduct disorder, mild intellectual disability, adjustment disorder, and reactive attachment disorder after adjusting for four parameters (sex, junior high school status, abuse history, and school refusal). Diagnoses present in three or more patients were analyzed. Explanatory variables that would affect the rate of the diagnosis were chosen.

Table 4 presents the results of multivariate logistic regression analyses between cases and controls for a diagnosis of ADHD and ASD with abuse history after adjusting for two parameters (sex and junior high school status). Explanatory variables that would affect the rate of diagnosis were chosen.

### Outcome data

No significant differences were observed in family history, depressive state, obsessive–compulsive symptoms, and diagnosis of mild intellectual disability between the two groups. Age, proportion of males, junior high school status, abuse history, and diagnoses of ADHD, ASD, conduct disorder, and reactive attachment disorder were significantly higher in cases than in controls. The proportions of patients with fathers and mothers as caregivers and with school refusal were significantly lower in cases than in controls (Table 1).

Multivariate logistic regression analyses revealed that sex, junior high school status, abuse history, school refusal, and diagnoses of ASD, conduct disorder, and adjustment disorder were independently associated with stealing. The proportions of males, junior high school status, abuse history, and diagnosis of ASD and conduct disorder were significantly higher, and the proportions of adjustment disorder and school refusal were significantly lower in cases than in controls (Tables 2 and 3).

The proportions of patients with fathers and mothers as caregivers and who were diagnosed with ADHD and reactive attachment disorder were associated with stealing by univariate, but not multivariate, logistic regression analysis (Tables 2 and 3).

When the two factors were considered together in Table 4, multivariate logistic regression analyses revealed that the proportions of patients diagnosed with ADHD and ASD with abuse history were significantly higher in cases than in controls (Table 4).

## Discussion

To the best of our knowledge, this case–control study may well be the first study in clinical settings to examine the clinical implications among child and adolescent psychiatric patients with or without a history of stealing.

With regard to the primary hypothesis, we observed that the proportion of patients with abuse history was significantly higher in cases than in controls. ADHD was the most common diagnosis in Table 1, followed by ASD as typical developmental disorders. The multivariate analysis revealed that the proportion of patients diagnosed with ASD was significantly higher in cases than in controls. The multivariate adjusted odds ratio increased further when the two factors were considered together, such as ASD with abuse history and ADHD with abuse history (OR = 7.81 and 4.46, respectively). Children with a history of stealing were more likely to be diagnosed with ASD or to have abuse history, or diagnosed with ADHD or ASD with abuse history. A previous study reported that a diagnosis of ADHD was predictive of oppositional defiant disorder (ODD). This relationship appears to be driven by symptoms of hyperactivity–impulsivity rather than inattention. ODD is also predictive of a diagnosis of conduct disorder [20]. In a meta-analysis and systematic review ADHD was associated with earlier onset and an increased risk of multiple offenses. The most frequently committed criminal offenses were theft, assault, drug- and weapon-related crimes [21]. The hyperactivity–impulsivity of ADHD and ASD may be related to stealing. A previous study [22] found that a broken home or disrupted family was predictive of delinquency, in agreement with the results of the present study. In this study, stealing was associated with psychobiological factors, such as ADHD, ASD, and abuse history. Although ADHD and ASD are not representative biological factors, the primary hypothesis was partly confirmed. In the presence of biological predisposition, such as developmental disorders, stealing may be characterized as an externalization disorder when psychological factors, such as a poor rearing environment, came together.

With regard to the minor hypotheses, cases were significantly older than controls (average age, 12.39 ± 1.93 vs. 11.03 ± 2.92 years, respectively). The average age of cases was >12 years, which is the age of junior high school in Japan. The proportion of junior high school students was significantly higher in cases than in controls. Although the relationship between ages after high school and stealing was not clear, the minor hypothesis was partly confirmed.

The results of multivariate logistic regression analysis confirmed that the proportion of ASD was significantly higher in cases than in controls (OR = 2.02). ASD is characterized by deficits in social communication and interactions, as well as restricted and stereotypical behaviors. In addition to these core symptoms, ASD in children and adolescents is often accompanied by behavioral problems, such as irritability and aggression, which may manifest as tantrums, self-injury, and aggressive behaviors toward others [23]. Although this result did not confirm causality of ASD and stealing by children, there was a correlation between outpatients with ASD and stealing. But as presented in Table 1, of the 326 children with ASD in this study, 16 (4.91%) reported a history of stealing at the first consultation. The lifetime prevalence of shoplifting in the USA is reportedly 14.1% for men and 8.8% for women [11]. It should be noted that the percentage in the present study was low as 4.91%, which meant children with ASD were not likely to steal something in general. Sprenger [24] reported that patients with ASD and additional symptoms of ADHD expressed a greater severity of autistic symptoms, especially in social interactions, as compared with those with ASD only. Among the cases in this study, of the 16 patients diagnosed with ASD, 10 (62.5%) had symptoms of hyperactivity and impulsivity. As mentioned above, symptoms of ADHD might have contributed to this result.

The proportion of conduct disorder was significantly higher in cases than in controls (OR = 49.5). The essential feature of conduct disorder is a repetitive and persistent pattern of behavior in which the basic rights of others or major age-appropriate societal norms or rules are violated. The diagnostic criteria of conduct disorder in the DSM-5 include stealing, which is described as “Has stolen items of nontrivial value without confronting a victim” [5]. Meeting the clinical diagnosis of kleptomania is rare due to age, as the prevalence in the general population is exceptionally low. Recurrent failure to resist impulses to steal leads to imprisonment. For such patients with these backgrounds, the psychiatrist might arrive at a diagnosis of conduct disorder when evaluating the causes of stealing.

The proportion of patients with adjustment disorder was significantly lower in cases than in controls (OR = 0.20). As the diagnostic criteria of adjustment disorder, the DSM-5 describes “The development of emotional or behavioral symptoms in response to an identifiable stressor(s) occurring within 3 months of the onset of the stressor(s)” [5]. A recent stressor might not affect the propensity for stealing differently from any other stressor, such as abuse history, which could become a psychological factor over time. This assumption is in line with the primary hypothesis that stealing is affected by psychological factors, suggesting that the long-term effects of psychological factors may be more relevant than any short-term effect.

In the present study, there was a higher proportion of males in cases than in controls (OR = 2.38). Among the cases, ADHD was the most common disorder, followed by ASD. Both ADHD and ASD are more common in males than in females [25, 26]. The prevalence and severity of childhood behavioral problems, especially rule-breaking behaviors, differ between males and females, as males tend to break rules more regularly [27], which might have led to the different results of younger vs. adult patients.

There was also a higher proportion of junior high school students in cases than in controls (OR = 3.40), in agreement with the results of previous studies, as stealing is more likely with older age [7]. The cases consisted of patients who self-reported or whose parents reported stealing. The extent of stealing by junior high school students, unlike elementary school students, should not be overlooked, as elementary school students may have a narrow scope of activity, indicating that their parents provide appropriate care.

In addition, there was a significantly higher proportion of abuse history in cases than in controls (OR = 3.73). It has been reported that sexual assault, child maltreatment, witnessing family violence, and exposure to other incidences of major violence independently contribute to the severity of both depression and anger/aggression [28]. Most adverse childhood experiences (ACEs) are strongly associated with three mental health outcomes, i.e., depressive symptoms, drug abuse, and antisocial behavior. Males who experienced ACEs are more likely to engage in antisocial behavior early in young adulthood than females who experienced similar ACEs [29]. This finding is similar to that of the present study; thus, it might be necessary to reconsider stealing as an externalization problem of children with abuse history.

There was a significantly lower proportion of students who refused to attend school in cases than in controls (OR = 0.35). School refusal has various causes, including bullying, which can result in emotional distress, depression, anxiety, social isolation, low self-esteem, school avoidance/refusal, and substance abuse of the victim as well as the bully [30]. Based on the finding that children with a history of stealing were likely to have abuse history, domestic stress might contribute to stealing rather than stress outside of the home, such as the school setting. As another viewpoint, children who stay at home without attending school were less likely to have a place to steal.

As mentioned above, the proportions of patients diagnosed with ADHD and ASD in addition to having abuse history were significantly higher in cases than that in controls (OR = 7.81 and 4.46, respectively). Among the cases, 11 patients were diagnosed with ADHD and ASD with abuse history. Among those with abuse history, including duplication, the proportions of physical (n = 5) and psychological abuse (n = 6) were higher than those of neglect (n = 2) or sexual abuse (n = 1). Of the 11 patients diagnosed with ADHD and ASD with abuse history, 8 (72.7%) started stealing when in elementary school (>12 years old), and 8 (72.7%) stole something outside of the home. Only 2 (18.2%) of these 11 patients had refused to attend school. These patients might express their heartache by stealing.

Finally, factors that were significant by univariate analysis but no longer significant by multivariate analysis were taken into account. After adjusting for sex, there was no longer a significant difference in the proportion of ADHD diagnoses. Statistical analysis also revealed that sex had the greatest impact on ADHD diagnosis, which is more frequent in males than in females in the general population, with a ratio of approximately 2:1 in children [5]. Hence, a diagnosis of ADHD is impacted by sex.

After adjusting for abuse history, there was no longer a difference in the proportions of patients with fathers and mothers as caregivers and who were diagnosed with reactive attachment disorder. The diagnostic feature of reactive attachment disorder in the DSM-5 is absent attachment between the child and putative caregiving adults due to limited opportunities of being given care during the early development [5]. Due to abuse history and separation from the caregiver, absent attachment occurs between the caregiver and child, thereby promoting the development of reactive attachment disorder [31]. Therefore, adjusting for abuse history might remove the significant difference in the proportions of patients diagnosed with reactive attachment disorder.

### Study limitations

This study had some limitations that must be considered. First, a diagnosis and information on abuse history were established after the first consultation. However, a diagnosis may change and additional information regarding abuse history may be included after further examinations. Inaccuracies were present in the detection of abuse history because many of the abuses were not immediately visible. In other words, we might have missed an abuse history. In this study, the diagnosis that best explained the current symptoms was adopted without consideration of comorbidities. Second, it is important to note that the results of this study confirmed not the causality but the associations between stealing and psychobiological factors. The factors assessed in this study were mentioned in previous studies, but there may be other unknown factors related to stealing. Third, this study had selection bias for several reasons as follows. The number of participants who had a history of stealing was only 56 patients. Due to the small sample size, results might be subject to random errors. This clinical study was conducted in a single district around Hachioji City. Also, this study did not explore the general situation associated with stealing among child and adolescent psychiatric patients. Nonetheless, there was a correlation between a history of stealing and older age. Lastly, we could not completely define the behaviors of stealing. The definition of stealing was based on self-reporting or reports from caregivers, and the final judgment as to whether the action was stealing or not depended on psychiatrists in charge. This study revealed a common clinical setting, but with limited consistency. Therefore, classification of cases into subgroups, such as targeting only psychiatric patients arrested by the police for stealing, may lead to more meaningful clinical findings.

## Conclusion

The proportion of patients with abuse history was significantly higher in cases than in controls, as was the proportion of ASD. The proportions of patients diagnosed with ADHD and ASD with abuse history were significantly higher in cases than in controls. The multivariate adjusted odds ratio increased further when the two factors were considered together, such as ASD with abuse history and ADHD with abuse history than that of each factor alone. Notably, this study is the first to confirm that children with a history of stealing were more likely to be diagnosed with ADHD with abuse history or ASD with abuse history. However, our study should be understood without prejudice because this study is reporting associations, not causality. Therefore, a prospective study to investigate causality among ADHD, ASD, abuse history, and stealing is needed. If ADHD and ASD with abuse history can be correlated to a history of stealing, interventions can be more effective by understanding the mechanisms underlying these connections.

## References

[1] Miller TR, Fisher DA, Cohen MA. Costs of juvenile violence: policy implications. Pediatrics 2001 1;107(1):E3 10.1542/peds.107.1.e3 11134467

[2] Loeber R, Hay D. Key issues in the development of aggression and violence from childhood to early adulthood. Annu Rev Psychol 1997;48:371–410. 10.1146/annurev.psych.48.1.371 9046564

[3] Tremblay RE. Developmental origins of disruptive behaviour problems: the 'original sin' hypothesis, epigenetics and their consequences for prevention. J Child Psychol Psychiatry 2010 4;51(4):341–367. 10.1111/j.1469-7610.2010.02211.x 20146751

[4] WHITE PAPER ON CRIME 2017, an annual publication of the Ministry of Justice, Japan

[5] American Psychiatric Association, 2013 Diagnostic and statistical manual of mental disorders 5th ed APA, Washington, DC.

[6] Goldman MJ. Kleptomania: making sense of the nonsensical. Am J Psychiatry 1991 8;148(8):986–996. 10.1176/ajp.148.8.986 1853988

[7] Barker ED, Séguin JR, White HR, Bates ME, Lacourse E, Carbonneau R, et al Developmental trajectories of male physical violence and theft: relations to neurocognitive performance. Arch Gen Psychiatry 2007 5;64(5):592–599. 10.1001/archpsyc.64.5.592 17485611 PMC3283575

[8] Nadeau MM, Rochlen AB, Tyminski R. The Psychology of Shoplifting: Development of a New Typology for Repeated Shoplifting. Int J Offender Ther Comp Criminol 2019 10;63(13):2338–2355.20 10.1177/0306624X19845979 31043101

[9] Blanco C, Grant J, Petry NM, Simpson HB, Alegria A, Liu SM, et al Prevalence and correlates of shoplifting in the United States: results from the National Epidemiologic Survey on Alcohol and Related Conditions (NESARC). Am J Psychiatry 2008 7;165(7):905–913. 10.1176/appi.ajp.2008.07101660 18381900 PMC4104590

[10] Vandereycken W, van Houdenhove VD. Stealing behavior in eating disorders: characteristics and associated psychopathology. Compr Psychiatry 1996 Sep-Oct;37(5):316–321. 10.1016/s0010-440x(96)90012-7 8879905

[11] Hoertel N, Dubertret C, Schuster J, Le Strat Y. Sex differences in shoplifting: results from a national sample. J Nerv Ment Dis 2012 8;200(8):728–733. 10.1097/NMD.0b013e3182613fbb 22850311

[12] Grant JE, Odlaug BL, Lust K, Christenson G. Characteristics and correlates of stealing in college students. Crim Behav Ment Health 2016 4;26(2):101–109. 10.1002/cbm.1986 26648022

[13] Grant JE, Potenza MN, Krishnan-Sarin S, Cavallo DA, Desai RA. Stealing among high school students: prevalence and clinical correlates. J Am Acad Psychiatry Law 2011;39(1):44–52. 21389165 PMC3671850

[14] Heath E, Kosky R. Are children who steal different from those who are aggressive? Child Psychiatry Hum Dev 1992;23(1):9–18. 10.1007/BF00706696 1424943

[15] Sourander A, Fossum S, Rønning JA, Elonheimo H, Ristkari T, Kumpulainen K, et al What is the long-term outcome of boys who steal at age eight? Findings from the Finnish nationwide "From A Boy To A Man" birth cohort study. Soc Psychiatry Psychiatr Epidemiol 2012 9;47(9):1391–1400. 10.1007/s00127-011-0455-8 22120609

[16] Barker ED, Tremblay RE, van Lier, Pol A. C., Vitaro F, Nagin DS, et al The neurocognition of conduct disorder behaviors: specificity to physical aggression and theft after controlling for ADHD symptoms. Aggress Behav 2011 Jan-Feb;37(1):63–72. 10.1002/ab.20373 21046606 PMC3283579

[17] Association AP (2000) Diagnostic and Statistical Manual of Mental Disorders, Fourth Edition American Psychiatric Pub 1 pp.

[18] Ministry of Education, Culture, Sports, Science and Technology: Survey on various issues in guiding students with problem behavior and school refusal (in japanese) http://www.mext.go.jp/a_menu/shotou/seitoshidou/1302902.htm

[19] Kanda Y. Investigation of the freely available easy-to-use software 'EZR' for medical statistics. Bone Marrow Transplant 2013 3;48(3):452–458. 10.1038/bmt.2012.244 23208313 PMC3590441

[20] Burke JD, Loeber R, Lahey BB, Rathouz PJ. Developmental transitions among affective and behavioral disorders in adolescent boys. J Child Psychol Psychiatry 2005 11;46(11):1200–1210. 10.1111/j.1469-7610.2005.00422.x 16238667

[21] Mohr-Jensen C, Steinhausen H. A meta-analysis and systematic review of the risks associated with childhood attention-deficit hyperactivity disorder on long-term outcome of arrests, convictions, and incarcerations. Clin Psychol Rev 2016 08;48:32–42. 10.1016/j.cpr.2016.05.002 27390061

[22] Wells LE, Rankin JH. Families and Delinquency: A Meta-Analysis of the Impact of Broken Homes. Soc Probl 1991 /02/01;38(1):71–93.

[23] Kaat AJ, Lecavalier L, Aman MG. Validity of the aberrant behavior checklist in children with autism spectrum disorder. J Autism Dev Disord 2014 5;44(5):1103–1116. 10.1007/s10803-013-1970-0 24165702

[24] Sprenger L, Bühler E, Poustka L, Bach C, Heinzel-Gutenbrunner M, Kamp-Becker I, et al Impact of ADHD symptoms on autism spectrum disorder symptom severity. Res Dev Disabil 2013 10;34(10):3545–3552. 10.1016/j.ridd.2013.07.028 23973801

[25] Sayal K, Prasad V, Daley D, Ford T, Coghill D. ADHD in children and young people: prevalence, care pathways, and service provision. Lancet Psychiatry 2018 02;5(2):175–186. 10.1016/S2215-0366(17)30167-0 29033005

[26] Werling DM, Geschwind DH. Sex differences in autism spectrum disorders. Curr Opin Neurol 2013 4;26(2):146–153. 10.1097/WCO.0b013e32835ee548 23406909 PMC4164392

[27] van der Sluis S, Polderman TJC, Neale MC, Verhulst FC, Posthuma D, Dieleman GC. Sex differences and gender-invariance of mother-reported childhood problem behavior. Int J Methods Psychiatr Res 2017 09;26(3). 10.1002/mpr.1498 PMC6877260 26799863

[28] Turner HA, Finkelhor D, Ormrod R. The effect of lifetime victimization on the mental health of children and adolescents. Soc Sci Med 2006 1;62(1):13–27. 10.1016/j.socscimed.2005.05.030 16002198

[29] Schilling EA, Aseltine RH, Gore S. Adverse childhood experiences and mental health in young adults: a longitudinal survey. BMC Public Health 2007 3 07,7:30 10.1186/1471-2458-7-30 17343754 PMC1832182

[30] Stephens MM, Cook-Fasano HT, Sibbaluca K. Childhood Bullying: Implications for Physicians. Am Fam Physician 2018 2 01,97(3):187–192. 29431974

[31] Hornor G. Reactive attachment disorder. J Pediatr Health Care 2008 Jul-Aug;22(4):234–239. 10.1016/j.pedhc.2007.07.003 18590868

